# Psychology of Leibnitz
†"Leibnitzii Opera Philosophica quæ extant omnia." Berlin. (By Professor Erdmann.)


**Published:** 1856-07-01

**Authors:** 


### Art. II.—PSYCHOLOGY OF LEIBNITZ.†

BY PROFESSOR HOPPUS, LL.D.

THE speculative science of the Germans must be regarded as dating from LEIBNITZ. The name of Puffendorf, indeed, is generally placed first among those writers who have contributed to the formation of an indigenous German philosophy; but he cultivated only one branch—jurisprudence. Leibnitz was in every sense universal. Our limited space obliges us to condense to the utmost as plain an account as we can give of the main psychological and metaphysical speculations, sometimes vagaries, of this great master-genius.

Gottfried Wilhelm von Leibnitz was born at Leipzig in 1646. He was contemporary with Newton, Clarke, Locke, Spinoza, Malebranche, and other celebrated men; and he was not eclipsed amidst the splendour of such a constellation—*micat inter omnes*. The period was favourable to the influence of such a mind: former polemic heats were allayed; and there was a growing taste for research, and for scientific intercommunication; witness the efforts, to this end, of Wallis in England, and Mersenne in France. Germany was prepared to take her part in this favourable crisis; and, in that country, Leibnitz's influence may be traced in almost every branch of speculation down to recent times. He, alone, is to Germany what Locke and Newton are to England. But his fame is European; and it is well remarked by Dugald Stewart, that his "best eulogium is furnished by the literary history of the eighteenth century." At school he was ardently devoted to classical learning; and he afterwards entered the University of Leipzig, in which his father was professor of

† "Leibnitzii Opera Philosophica quæ extant omnia." Berlin. (By Professor Erdmann.)

jurisprudence. Here, though he studied for the law, he applied himself intensely to almost every branch of knowledge, but especially to mathematics and philosophy; and, in pursuit of the latter, he devoted much time to Plato and Aristotle. He early formed the project of laying the foundation of a new and improved system of knowledge, in the attempt to combine whatever he could harmonize in the views and methods of these ancients, with those of Descartes. His prodigious memory enabled him to retain and recal his immense reading, but did not damp his ardour for new discoveries, and for intercourse with all the great men of his day who came within his reach. Almost fabulous statements are made of his vast industry; but, no doubt, in this, as in other things, he surpassed ordinary mortals, though it must be an exaggeration to state, as Fontenelle does, that he "did not leave his study-chair, day or night, for weeks together." So anxious was he for knowledge of all kinds, that he was ready to identify himself, for the sake of it, even with pretenders or fanatical devotees to science. When still in his youth, he joined a society of alchemists, at Nüremberg; into which he craved admission by writing a letter to the adepts, which was so appropriately obscure and mystical as to induce them eagerly to elect him as their secretary. His more public literary and scientific career may be dated from the time when he was persuaded to withdraw from this confraternity by the Baron von Boineburg, chancellor of the Elector of Mentz, and whose valuable patronage he now obtained.

The amount of Leibnitz's writings is immense; yet throughout all his speculative pieces one idea reigns—that of a *prima philosophia*, a science of first principles, which is to be the basis of all systematic knowledge. But though his aim was thus one and unique, his writings were for the most part inspired by occasions, and were miscellaneous, often of a fragmentary character, found in reviews and detached pieces, or in his extensive correspondence. All are constantly marked by the same tendency to general principles and fundamental truths, and to the harmonizing of previous and apparently discordant systems, ancient and modern—by the fertile and inventive character of his imagination, and by his inclination to introduce mathematical ideas into general subjects. By adopting demonstrative methods in the psychological and moral sciences, he sanguinely hoped to put an end to controversy. In thus maintaining that the methods and spirit of mathematics should be carried into all other studies, he symbolized with Descartes and Spinoza, however much he differed from them in other respects. He first distinguished himself in 1664, on taking his Master's degree, when about eighteen years of age, by his inaugural thesis *de Principio Individuationis*, in which he shows an accurate acquaintance with certain phases of the scholastic philosophy, and takes the side of the nominalists in regard to the question of universals. He also here claims a real positive existence for individual objects, contrarily to the views of Spinoza; and sustains his opinions by appealing to Aureolus, Gregory Arimensis, Gabriel Bird, Durandus, and others, whom he either quotes or refers to. We must not attempt to give a particular account of his treatises, which now followed each other in quick succession; and which, from first to last, compassed almost the entire circle of human knowledge. We can only touch on a few of these pieces—chiefly those which bear more closely on psychological matters. His "Ars Combinatoria," 1666, and his posthumous fragment entitled, "Historia et Commendatio Linguæ characteristicæ universalis," both relate to an idea which he conceived when 16 years of age, of framing an alphabet of human thought, which should comprise the most simple elements of our ideas, and express their combinations. There was some analogy between this scheme and some recent methods* which propose to reduce reasoning, or even thought in general, to an algebraical calculus. Leibnitz was astonished, he tells us, that such a method had escaped Aristotle and Descartes: but he never followed it out into the actual construction of a universal language. It is curious, that about this time, our philosopher employed his logic to persuade the Poles to choose the Prince of Neuburg to their elective monarchy; but though he brought into the field a phalanx of sixty propositions, bristling with axioms rigorously applied, his logic was not strong enough to seat the prince on the throne.

Leibnitz was now rapidly rising to honour, and he was made a councillor and chancellor of justice to the Elector of Mentz: but he still thirsted after knowledge; and his next project was an Encyclopædia of learning, on the basis of that of Alstedius: however, this, like many other gigantic enterprises which he conceived, was never realised. Amidst his other works on law, politics, history, philosophy, and two on mechanical principles—which latter were rejected by men of science as resting on no solid basis,—we see a "Defence of the Doctrine of the Trinity, on the principles of Logic"!† Soon afterwards we find Leibnitz inventing an arithmetical machine, intended as an improvement on that of Pascal, and which is still to be seen at Göttingen. He rejected, about this time, the place of Pensioner of the French Academy, as it would have involved his professing the Catholic religion; yet this discursive, and sometimes eccentric genius, in a work on German diplomacy, actually propounded a

* Particularly by Professors De Morgan and Boole.

† "Sacrosancta Trinitas per nova argumenta logica defensa," 1670.

scheme for making the pope the head of the Protestant as well as the Catholic Church in the west, the German emperor being the co-ordinate civil ruler. The idea was that of a general government for Europe. Leibnitz actually corresponded with Bossuet on the subject of a Catholico-Protestant union, on the principle of mutual concessions. A year or two afterwards we have a new phase of our author in his "Protogæa," a work on the formation of the earth, and of which Dr. Buckland has recorded that it contains germs of the most enlightened speculations of modern geological science. In 1710 appeared Leibnitz's celebrated work, "Essai de Theodicée," in which are contained his doctrines of pre-established harmony and optimism. It is, in short, a defence of the wisdom of God, viewed in connexion with the existence of evil; and it was aimed against the atheism of Bayle. His "Monadologie" was posthumously published, and was composed for Prince Eugéne of Savoy. Just before his death, Leibnitz was engaged in the celebrated controversy with Dr. Samuel Clarke, on freewill, the reality of space, and other knotty points, which were all discussed in a series of letters, in 1715 and 1716; in which great learning is exhibited on both sides, with occasional sharpness of retort on the part of our philosopher. His important work, "Nouveaux Essais sur l'Entendement Humain," was not published in his life-time. It was written as early as 1704, in answer to Locke's Essay; but Leibnitz did not publish it, on account of Locke's death: "for," (said he in a letter to Remonde) "I cannot bring myself to publish refutations of deceased authors."

The fame of Leibnitz during his lifetime was perhaps rarely equalled. Even crowned heads did him honour. The German Emperor awarded to him a pension of two thousand florins, with the dignity of Baron of the Holy Roman Empire, and Aulic councillor; and the Czar Peter conferred on him the title of privy-councillor with a pension, in return for his advice in regard to the civilisation of the Russian people. His talents were undoubtedly great, and his ambition was greater. He even tried his hand at poetry; but, like Cicero, he did not find the muse propitious, and he gained no laurels by her inspiration. He wrote a piece* in recommendation of the study of his native tongue, but he composed very little in it. Frederic Schlegel remarks that "it is a pity that he did not write his philosophical works in German: it would have been impossible to have left so many divinely illuminated thoughts to swim in such a sea of sciolism (halbheiten) as, alas! has often happened in the barbarous scholastic Latin, and in French."† It was half a century

* Vid. "Collectanea Etymologica," Dutens, vi. p. 651.

† "Einleitung zu Lessing's Gedanken."

later before German assumed the position which it now holds among the languages of Europe. Some of Leibnitz's remarks show that he himself was not fully aware of its resources and its flexibility.* His Latin style is not generally elegant or pleasing, and it often contains Gallicisms. It was not, on the whole, such as to expose him to be beaten by the angel, as was said of one of the Fathers, for writing with the pen of Tully. It is less remarkable that his French prose should occasionally fall into Germanisms; but his French style is not wanting in simplicity and force, and it is often marked by a great combination of life, point, and beauty.

There has never, that we are aware, been a complete edition of Leibnitz's writings. Probably no one has ever waded through all the vast heterogeneous matter that has actually seen the light. Dutens's edition (Geneva, 1768) is in six quarto volumes, of from 600 to 1000 mortal pages each; but it is far from containing all. Raspe published an important collection, entitled "Œuvres Philosophiques de feu M. Leibnitz." In 1805, Feder added a selection of letters never before printed. In 1840, Professor Erdmann, of Berlin, published "Leibnitzii Opera Philosophica quæ extant omnia," in one volume, which is a much more complete collection of his pieces on speculative philosophy than is to be found either in Dutens or Raspe. In 1845, M. Jaques published a selection from Leibnitz's philosophical writings, at Paris. Our author has had many biographers, of whom the most recent is Guhrauer, who has made valuable additions to our knowledge of this great man. We have some curious autobiographical fragments, in which Leibnitz describes the development of his own mind. He here tells us how he invented for himself an original method of studying Latin. He began by "devouring Livy, and the Chronological Thesaurus of Calvisius." The latter he easily managed, because he had a German book on the same subject; but Livy required to be "devoured" *secundum artem.* He at first chiefly pored over the words immediately under the wood-cuts, and, at all events, missed everything which seemed obscure and difficult. This operation he repeated several times, and every time understood more and more, till at last almost all was plain, and this "without a dictionary."

He also informs us how his master wanted to check his ardour, on pretence of regulating his studies, but really on account of his own ignorance; how he read Latin pretty well, knew some Greek, and made verses, at twelve years of age. Logic seems to have been next attacked, and our author says

* "Œuvres de Leibnitz," Dutens, v. 331.

that while others were horrified at this study, he was delighted with it. He tells us that his

"Stature was of the middle height, and graceful; that his face was pale, his hands generally cold, his sight keen, his voice shrill rather than powerful, and that he found some difficulty in pronouncing the guttural letters, but especially the *k*; that he went late to bed, and greatly preferred night studies; that he read much from childhood, and thought more: that in most things he was self-taught (αὐτοδίδακτος); he was endowed with an excellent invention and judgment, and found no difficulty in transferring his attention rapidly from one subject to another, successively reading, writing, and speaking, and investigating any matter to the very bottom: he was neither very sad nor very merry, alike moderate in joy and grief, and he more frequently smiled than laughed."

Leibnitz died in 1716, in his seventieth year.

Ludovici and Hanscius have severally attempted a digest of the philosophical opinions of Leibnitz, but neither of their treatises is to be found in our British Museum. Such a work, if well executed, would be of great value in abridging the heavy toil of the student. The Protean forms which the genius of Leibnitz assumed, the fragmentary manner in which he has treated many subjects, and the immense accumulation of materials and of reflections on them which abound in his writings, are often quite bewildering, as it is necessary to pursue his opinions on some topics over so vast a field, in order to do them justice. After all, the reader must often content himself with general principles and germs of thought, which the author, impelled onward by his ardour to embrace everything within the sphere of human knowledge, did not allow himself time fully to systematize and digest. Like Descartes, he aimed at a philosophy which should embrace the first principles of all truth, by a method as nearly as possible like that of mathematicians. This was his ruling idea, and he was ever seeking, by means of it, to give a certain unity to the ever-changing and kaleidoscopic phases of his invention. With Spinoza he set out from Cartesianism; but, from the first, Leibnitz holds more of the objective than Descartes, whose dualism did not satisfy him, though he preferred the ideal tendency of this great modern founder of more speculative intellectual inquiry to the Lockian principle that we, in some way or other, owe all our knowledge to experience. "Descartes," said he, "has not led us into the innermost apartment of truth, but he has led us into the antechamber."* He praised Descartes' work "De Methodo," and his "Meditationes," as being in the spirit of Plato, withdrawing men's minds from sense to thought, usefully reviving

* Op. ii. p. 263, Dutens.

the ancient academic scepticism or caution ; but he justly charged Descartes with speedily departing from his own principles, and quitting doubt for hypothesis. Leibnitz, therefore, aimed at a more consecutive and consistent method. We must seek for its illustration, and for the opinions of the great Coryphæus of German speculation in general, through the mass of his occasional papers, such as his contributions to learned societies and his correspondence, as well as in his fuller and more systematic treatises, as his " Nouveaux Essais," and his " Theodicée," though even here we look in vain for a formal and complete detail, or even an outline of a system of philosophy. We are therefore compelled to frame to ourselves some order in which best to view his opinions, and to exhibit them briefly in their connexion and mutual dependence.

It is proper to glance first at his doctrines relating to the NATURE, ORIGIN, and CERTAINTY of HUMAN KNOWLEDGE, as these points closely involve the prime psychological and logical operations of the mind ; and, as in all the great writers who have influenced philosophy on the side of psychology, their main respective views on the grounds and method of knowledge, have always been characteristic and fundamental. Thus Bacon laid the basis of knowledge in experience by intuition. Descartes began with doubting, and placed certainty in that of which we could not doubt. Spinoza identified human and divine thought, and held them to be the ground of knowledge. Malebranche reduced all truth to " vision in God." Berkeley found certainty only in our ideas,—all else was illusion. Hume held no higher truth than association ; with him causation involved no positive and universal necessity. Locke traced certainty to the agreement of our ideas, as derived from sensation and reflection. Hobbes, Gassendi, and Condillac based it ultimately on sensation alone. Leibnitz, like Descartes and Spinoza, endeavoured to reduce the form of truth and knowledge to rational axioms, though he tested them in a different way. His views on the nature, origin, and certainty of our knowledge may be gathered from his tracts and fragments, entitled " Meditationes de Cognitione, Veritate, et Ideis," " De Veritatibus Primis," " Definitiones Logicæ," " Difficultates quædam Logicæ," " Schreiben an Gabriel Wagner vom Nutzen der Vernunftkunst oder Logik," " Reflections sur l'Essai de M. Locke," " Discours touchant la Méthode de la Certitude," " Nouveaux Essais sur l'Entendement Humain," and elsewhere. All the above, with the exception of the " Nouveaux Essais," are brief pieces.

Leibnitz's theory of knowledge is in its spirit Cartesian, or, as he himself would say, Platonic, as aiming at the *à priori* method rather than that of experience. The fact is, that neither method should be attempted alone; they should correct and limit each other. Of the two, however, we doubt not that the "high *à priori* road" is the most dizzy and dangerous. It easily led Pythagoras to *number* as the essence of all things; it conducted the Eleatic school to Pantheism: Plato into the mystic region of the Supersensibles; Descartes to his plenum of vortices: it led Malebranche to transfer the objectivity of our perceptions from the sensible world to the Deity; and Spinoza was conducted back by it to a semi-eleatic pantheism, and this by the march of what has been called a "pitiless logic," though with a sufficiently *pitiful* result. The difficulty is to distinguish, in axiomatic assumptions, what is rational and necessary from what is merely egoistic and fanciful. Though Leibnitz had some admirable notions on first principles and axiomatic truths, he also fell a victim to dogmatic hypothesis,—witness his romance of "Monadology," which he appears really to have believed. The *à priori* method, unrestrained by the modesty of reason and the due sense of the limitation of the human faculties, has in subsequent times produced the vagaries of Fichte, Schelling, and Hegel, and other popular systems in Germany. Granting, as we do, the value, importance, and necessity of fundamental or unprovable truths, in their proper sphere, we only mean that the greatest possible caution is necessary in the details. Leibnitz himself, with all his genius, was eminently at fault in his appreciation of the legitimate scope of the human faculties.

In the outset, however, of this theory of knowledge he is more cautious than his predecessor Descartes, who held that everything is true which is represented to us with perfectly clear ideas. Leibnitz calls for such a test of this clearness as can be relied on for truth; and finding none, he prefers the rules of logic as more suited to be a criterion. What he means by logic, he distinctly tells us in his letter to Wagner. He would not limit it to the testing of truth, but would make it include methods of discovery; in other words, he would embrace within its sphere both deduction and induction.* Leibnitz distinguishes the qualities of our ideas as *clear*, when we can ourselves distinguish them; *distinct*, when we can accurately describe their difference to others; and *adequate*, when all the distinctive marks are equally capable of being described. The three marks are found together in but few of our ideas; for we rarely know for certain that we have reached their final elements in the analysis. When, however, this is the case, our knowledge is *intuitive*, or *à priori;* it is derived, says Leibnitz, not from the

* "Unter der Logik, verstehe ich die Kunst den Verstand zu gebrauchen, also nicht allein was fürgestellt zu beurtheilen, sondern auch was verborgen ist zu erfinden."—Schreib. an Wagn.

senses, but from the intellect itself. He instances our ideas of number, as capable at once of a clear, distinct, and adequate (perfect) analysis, and as therefore intuitive.* He also would say, that when we can thus attain to perfect knowledge, we have a "*real* definition;" in all other cases only a "nominal" one.† The pure mathematical sciences are the region of these adequate and intuitive ideas. It is impossible for us here to dwell on the vexed controversy of *logical definition;* we merely state in brief Leibnitz's distinctions. Again, an idea is "true," as such, when it is *possible;* "false," when it involves a contradiction. Possibility is known *à priori*, when the ultimate elements of our ideas contain no incompatibilities. "Pure intuition of the primary possibles, by the resolution of ideas to their irreducible elements, is the source of all absolute and demonstrative truth." In plainer language, logical truth depends on the compatibility of the hypotheses which lead to it; but the Cartesian, Leibnitzian, and other continental speculations regarding "possibility" and truth, we hold to be often fanciful and mystical, and not always intelligible. We know possibility, *à posteriori*, on the other hand, continues Leibnitz, by actual existence; for if a thing exists, it must be possible. The above distinctions were partly brought forward by way of amending Descartes' principle, that "whatever I clearly and distinctly perceive of anything is true," —a principle, like most others, far from being exempt from licence and abuse, though designed to be final. Leibnitz's attempt to rectify or supplant it, though somewhat more critical than the principle of Descartes, equally takes for granted that all the criteria brought forward can always be depended on, and precisely and strictly applied by human sagacity. But the infirmity of reason was not one of those facts which were most apt to dwell on the sanguine and adventurous mind of Leibnitz: he was far more habitually influenced by a conviction of the importance, in some way or other of hitting upon a remedy for the imperfection of the existing methods of philosophy; and his reigning idea, as to this panacea, was the reduction of all our knowledge by means of the *à priori* process, as found in mathematics.

It may be well to observe, that our author's general views respecting the nature of "*ideas*" were perfectly simple, and untinged with any infusion of the ancient idealism, or of its relics in later times. He was not more a Platonic or mystical idealist like Malebranche, or a spiritualistic idealist like Berkeley,‡ than he was an idealist of the Peripatetic school; he distinctly rejected

* "Meditat. de Cognit.;" "Nouv. Ess."

† Ibid. "Reflections sur Locke."

‡ Vid. our account of Berkeley's Psychology, in this Journal, July, 1855.

all sorts of images, and all their shadowy resemblances, and he pronounced our ideas to be nothing more than certain affections, or modifications of our minds. Descartes also had clearly maintained that no material analogies can explain thought; his "ideas" were, in like manner, only conditions of our minds; but in proposing to deal with ideas, according to mathematical procedure, he differed from Leibnitz, inasmuch as he greatly neglected logic as the source and type of deductive truth. It was by way of facilitating the application of strictly logical processes to our knowledge that Leibnitz attempted so much to elaborate a theory of ideas. To this end he brings his views to a point, when he lays down, as an *à priori* test of all deductive truth, the principle of *contradiction;* which Aristotle had long ago termed the "most certain of all principles" (βεβαιοτάτη τῶν ἀρχῶν πασῶν,) and announced in the form: "a thing cannot be and not be at the same time." A familiar instance of this principle of contradiction is found in Euclid's Problem, "to find the centre of a given circle," which is solved by *reductio ad absurdum,* as the principle of contradiction is commonly termed when applied to mathematics. But there is another principle, that which embraces the real and the actual—all the things among possibles, says Leibnitz, which the Deity has caused to pass into positive existence. This is the principle of the *sufficient reason;* and it includes the law of fitness or order, the law of causation, and of final causes: these are only various expressions or formulæ of the general principle.

Leibnitz describes the two principles in his "Monadologie," first published from the original French manuscript by Erdmann, in his recent edition of Leibnitz's philosophical writings; the Latin translation of this manuscript being entitled, "Principia Philosophiæ," in the edition of Dutens. "By the principle of contradiction we judge to be false what opposes the true, and true that which opposes the false: and by the principle of the sufficient reason we consider that no fact can exist without some sufficient reason why it is so, and not otherwise; though the reason itself may most frequently escape our knowledge." The first principle relates to the truths of *reason,* which are necessary, and their opposite impossible, as involving a contradiction; the second principle relates to the truths of *fact,* which are contingent, and their opposite hypothetically possible. In analyzing mathematical deductions, we come at last to primitive or identical propositions, the opposites of which contain contradictions; as in the case of $(a+b)(a—b) = a^2—b^2$; respecting which kind of propositions Aristotle directly taught that equality is identity (ἐν τούτοις ἡ ἰσότης ἑνότης). But while rational truths may be reduced to identities, and their denial involves contradiction, facts, or contingent truths belong to the principle of the sufficient reason. "There is," says our author, "an infinity of movements and inclinations, present and past, which enter into the efficient cause of my writing at this moment; but there must be a sufficient or final reason or cause of it, out of the series of contingencies; and this is God." Ultimately, Leibnitz reduces even the principle of contradiction to that of the sufficient reason, and with more propriety, as appears to us, than Wolf, his successor sought to reduce the latter to the former; for it is certainly a sufficient ground for believing demonstrated truths, that their denial involves contradiction. We doubt, however, the propriety of Leibnitz's extending his idea of the principle of contradiction to those axiomatic truths which Kant afterwards termed "synthetic judgments *à priori*," as for instance, *every change must have a cause;* for we can hardly reduce the denial of this to a logical contradiction, as in the case of Kant's "analytical judgments," in which the predicate is only an exposition of the subject, not a new idea. To deny that *all matter is extended*, no doubt involves a contradiction in terms; for matter means extended substance; but we believe in causation simply because we cannot help it, whether this be held to be a "sufficient reason" or not.

As to the principles themselves—that which can be reduced to identity is true, and cannot be contradicted without absurdity—and everything which happens in the universe of mind or matter, involves some adequate cause (sufficient reason) why it did not happen differently, whether we *understand* that reason or not—our author justly regarded these two principles as requiring no proof, being the spontaneous dictate of human intelligence. We think him right, also, in maintaining that, without the principle of the sufficient reason, we could not arrive at the proof of the existence of God.* It is equally plain, he adds, that "every part of mathematics may be demonstrated on the principle of contradiction or identity; and that in Natural Theology, and Natural Philosophy (so far as the ultimate principles of dynamics are concerned) we find the sphere of the sufficient reason. It was only thus that Archimedes, in his book 'De Equilibrio,' could account for the fundamental property of the balance."

The principle of the sufficient reason was the starting point of Leibnitz's controversy with Dr. Samuel Clarke; and it was mixed up with the question regarding the determination of the divine will. Clarke admitted that nothing *is*, without a sufficient reason why it is as it is; but, he added, that the mere will of God is often the only sufficient reason, in such a sense, that unless the will of God is capable of acting without a predeter-

* "Recueil de Diverses Pièces."

mining cause, his power of choosing would be reduced to mere fatalism. Leibnitz replied, that Clarke only granted the principle in words, but in reality denied it, and involved the conclusion that God wills some things without a sufficient reason for so doing. The dispute was, in fact, the old one about liberty and necessity—one which we apprehend will never be settled satisfactorily by argument. In this controversy many of Leibnitz's theories on other subjects came incidentally forward; among the rest, his definition of time and space as things merely relative, the former being the "order of succession," and the latter, the "order of coexistence." And this surely is all that can be said, *objectively*, of these two mysterious substrata of all our knowledge. His doctrines of monadology and pre-established harmony also incidentally appear in this controversy.

With regard to the relation of the Divine Mind to truth, Leibnitz maintained that all *possible* truth dwells in the understanding of God, and is, like himself, necessary and eternal; *actual* truth, or that which *is*, depends on his will. Matters of fact are contingent, like the laws of nature, on which they depend: they might have been different from what they are; or at all events they have only a moral necessity, that of order and fitness—a necessity arising only from the certainty that God will make the best possible choice, and not to be confounded with absolute mathematical and metaphysical necessity, which attaches only to the truths of reason. The sphere of absolute necessity includes metaphysics, logic, ethics, and mathematics; the sphere of actuality and reality includes physics in all its branches. Hence two modes of inquiry; logical deduction on the one hand, and experimental induction on the other.

An important point of our author's general theory of knowledge relates to the question of the *origin of our ideas*. Different schools are characterized by their respective views on this subject. In regard to a vast mass of our ideas, there is and can be no controversy. Kant on the one hand, and Locke on the other—not less than the materialists, who exaggerated and metamorphosed Locke's doctrines, as the German Pantheistical idealists did Kant's—agree that experience alone can teach us the properties of things around us. But it is evident that we have knowledge which transcends all experience, otherwise we could never believe irrevocably in the necessity and universality of causation. We shall not repeat here what we have said on the subject of "innate ideas," in our notice of the "Psychology of Descartes" in a former number.* "Innate ideas," and "innate principles," are unfortunate expressions, intended by the school of Descartes and by Leibnitz, to mean nothing more than ideas and prin-

* Vid. "Psychological Journal," January, 1855.

ciples which are only *occasioned* by experience, but which really emanate from the constitution of the human mind itself. We have further explained this subject in the "Psychology of Locke," in another number of this journal.* Locke evidently did really admit the very doctrine which he appeared to deny, when he argued at length against "innate truth." He was only combating, throughout, an exaggerated form—we may almost say a caricature, of the Cartesian view of "innate truth;" and he did not sufficiently distinguish between logical propositions, of which the "children" and "savages" whom he adduces "know nothing," and those psychological impressions, activities, and tendencies (Leibnitz would term them, "virtualities") on the basis of which they exercise their mental faculties without being aware of it. There is every reason to believe that, had Locke survived—who died just at the time when Leibnitz was about to publish his "Nouveaux Essais," in which he follows Locke's Essay throughout, in its divisions, chapter by chapter—a most instructive controversy would have arisen between these two great psychologists, equally useful to them, mutually, and to the world at large. We have no doubt that the issue would have been a far greater agreement of opinion on the subject than the uninitiated reader of Locke's dissertation on "innate ideas" would have supposed possible. There are not a few passages in the "Essay" which might very well have been penned by Descartes or Leibnitz himself; for they admit, in reality, all that these two philosophers would, on explanation, have contended for, though in a less technical—perhaps, in some respects, a less objectionable, phraseology.† Lord Shaftesbury, in his "Characteristics," put the question in the right light, when he said: "Is the constitution of man such, that, sooner or later, certain ideas will infallibly, inevitably, necessarily, spring up in him?"

Leibnitz, however, like the Germans generally, and like some of our own countrymen who, without sufficient caution, follow in their wake—failed to apprehend the scope of Locke, when he represented him as maintaining the scholastic principle, *nihil est in intellectu quod non fuerit prius in sensu.* This charge is strictly applicable to the sensational theory of the "ideologists," as well as to that of the decided materialists: it answers as a description of the tenets of Gassendi, Hobbes, and Condillac; but it certainly does not fairly apply to Locke, who distinctly held that there was "another great source of ideas besides sensation, namely, *reflection,* by which the mind furnishes the understanding with ideas of its own operations."‡ Nor did Leibnitz very cleverly *mend* the aphorism of the schools by

* Vid. "Psychological Journal," July, 1854.

† Vid. "Essay," Bk. iv. chap. 11 and 13.

‡ Ibid. Bk. ii. chap. 1.

appending to it "*nisi ipse intellectus*," as though, in any tolerable sense, the understanding could be "in itself." What he meant, however, was plain; and Locke better expressed it by saying, that reflection must be added to sensation as a source of knowledge; for, admitting fully the doctrine of primary truth as a general principle, it is, after all, only by self-conscious reflection that we come to notice the *à priori* constitutional tendencies and operations of our minds. Leibnitz's views of the origin of human knowledge were essentially taken from the stand-point of Descartes; and his theories on the subject were so much cast in the Cartesian mould, that it is no wonder he should, on the first reading of Locke's unfortunate chapters on "innate ideas and principles," feel disposed to speak very disparagingly of the Essay, as he does in his short tract on it, in 1696. Scarcely any two men could be supposed with more differently constituted minds than Leibnitz and Locke. In this tract, the former saw no difficulty in arriving at the conclusion that the soul *must* ever be active, and "always *think*." Locke, with his usual caution, did not deny this; but only said, that as we are not conscious of it, we want the evidence of it. Leibnitz, too, spoke more familiarly of the "infinite and the absolute" than Locke could do; and this fashion has been very current among the later Germans of the ideal school, and some of the French eclectics. M. Cousin would seem to have almost entirely got over the difficulty of comprehending the "infinite," if we may judge from some of his strange assertions with regard to our knowledge of the Deity. In the "Nouveaux Essais," Leibnitz speaks with great respect, and even eulogy, of Locke, and in a tone little in harmony with the flippant remarks on him which have escaped some of the Germans and French, and even some of his own countrymen, who ought to have understood him best. We have no doubt that Leibnitz was right in maintaining that certain ideas, such as cause, existence, etc., are elicited only, but not *formed*, by experience; and that certain truths, as the axioms of geometry, for example, are universal, necessary, and constitutional to the human mind, *psychologically*, previous to their being framed into logical propositions. Locke observed that the given ideas arose in connexion with experience, and that the alleged truths were not *logically* familiar to all minds; and, therefore, he hastily rejected, altogether, the theory of what are (by a term very liable to be misunderstood) called "innate" principles, in the modified and unobjectionable sense of Descartes and Leibnitz. Yet Locke really did admit these very principles, in fact, apparently without heeding it, as we have shown in our notice of Locke's psychology already alluded to. Leibnitz sometimes appears delighted with Locke's statements, quoting them at length, or alluding to them, as closely approximating in substance to his own views of "innate" truths, which he sometimes terms "natural virtualities." "It appears," says he, "that our able author (Locke) assumes that there is nothing virtual in us: but we must not take this rigorously." Leibnitz again remarks: "I am inclined to believe that, fundamentally, his sentiments on this point are not different from my own."

In respect to our idea of God, Leibnitz's views are Cartesian. But, here, we must observe that he appears to have fallen into a singular contradiction on this subject. He makes Théophile, who always represents his own opinions in the dialogue, say to Philalèthe: "I have always held with the innate idea of God as Descartes maintained it; and I am still of the same opinion.* But Philalèthe, having occasion, afterwards, to name the principles which Lord Herbert of Cherbury laid down as innate, says that some persons denied them. To this Théophile replies, that while he takes all necessary truths to be innate, he maintains that "these propositions are not innate, for they may be proved."† Now, one of Lord Herbert's "innate propositions" is, that "there is a Supreme Deity." We find no clue to any solution of this inconsistency of statement into which Leibnitz has fallen. We must not dwell further on this remarkable incongruity: we can only add, here, that, in our view, it is more accurate to say that the idea of *causation* is innate, than that the idea of God is so; though the necessity of causation, which is the same thing as adequate causation, evidently leads us, by one single step, to an Author of the universe. We hold that what are distinguished, technically, by the Germans, as the "ontological," "cosmological," and "physico-theological" arguments for a Deity,‡ are, in fine, only so many forms of the argument from causation.

Having given some account of the views which may be found, respecting the theory of human knowledge, scattered over various parts of Leibnitz's works, we shall now briefly sketch his MONADOLOGY; which was written at the desire of Prince Eugéne, and first published under the title of "Principia Philosophiæ." Agreeably to the Baconian ideas, so much in harmony with the English mind, our distinguished countryman, Locke, indulges in no speculations as to the nature or essence of soul or mind; and we may add, the nature or essence of matter. Leibnitz, on the other hand, brought for the solution of both these questions a

* "Vous savez, Philalèthe, que j'ai toujours été, comme je suis encore, pour l'idée innée de Dieu," etc.—"Nouv. Ess." chapit. i.

† "Je vous avoue que ces cinq propositions ne sont pas des principes innés, car je tiens qu'on peut, et qu'on doit les prouver."—Ibid. chapit. ii. 15.

‡ Vid. Kant's "Kritik." (Rosenkranz, 1838), s. 462, seq.

bold and we may say fanciful theory, of which he appeared much enamoured, though his followers have disputed respecting the meaning of not a few of the dogmas which he lays down; and some have gone so far as to assert that he intended the whole as only a flight of imagination. Of this, however, we have met with no evidence, and we see no reason to doubt his sincerity, though very much cause for more than doubting his judgment, in propounding this philosophical romance. His monadology, however, stands in very close connexion with his views of the nature of knowledge, and especially with its *à priori* aspect. For if he had not exaggerated the power of the mind to seize on truth intuitively, and to arrive at it by abstract thinking, he never would have been so far led away as to suppose that reason could construct all the elements of a universe by diving into the depths of consciousness. In this and some of his other theories, Leibnitz shows, by anticipation, the true general type of that idealism which has so largely been exemplified in the modern German mind. We do not mean to say that no truth is mixed up with his monadological speculations; we speak of the theory as a whole, and of the gratuitous assumptions which are often admitted into it, without any proof, or the possibility of proof to the capacities of man: yet our sanguine author boldly marches forward through all difficulties, and where to the eye of ordinary mortals there seems no road or clue to any safe conclusion, he easily finds one, and pioneers his way like a giant striding through forests and over mountains, as readily as other men can walk on plain ground.

The simple, says our philosopher, is that which has no parts—therefore no extension, therefore no form, therefore no divisibility. Such is each *monad;* and monads are the true atoms, unities, and elements of all things, whether material or intellectual. At first Cartesian in his views of matter (as essentially so in other respects, in his method) he identified matter with extension. But now he argued strenuously against this most defective theory of Descartes, as may be seen in various of Leibnitz's treatises.* Even resistance ("antitypie") added to extension does not, says he, explain motion; we must also have a force of elasticity. He traces the pantheism of Spinoza (which he terms a "detestable doctrine") and the hypothesis of "occasional causes," as held by Geulincx and Malebranche, to this dogma of Descartes—that matter and extension are one and the same: for, "if so, all things are but fleeting manifestations of one permanent substance, the Deity, as identified with nature;

* "De Verâ Methodo Philosophiæ;" "Epistola ad Thomasium;" "Commentarius de Animâ Brutorum; "Lettre à Foucher;" "Epistola ad Des Bosses;" "De la Nature en elle-même;" "Lettre à Bourguet," etc.

and as mere extension is incapable of action, we must at once have recourse to the agency of God as the immediate source of all motion. There must therefore be a *force* in things. The mere succession of phenomena does not meet the case; we must suppose a dynamic power—a cause or force, which we conceive of by an irreducible ("innate") idea. We are compelled to this view by the principle of the sufficient reason." But our author went farther: each monad is a force existing not in *posse* only, but also in *esse*. It is always more or less acting, like a bent bow or a suspended weight; indeed, we find, in these very cases, that the monads are always striving to change their condition, because each is a force, ἐντελέχεια, a term used by Aristotle for an efficient cause. Extension is the basis of geometry; force of dynamics. Matter, however, has not only this active power or force—it has also that passive power of resistance which Kepler called *inertia*. Leibnitz identifies *inertia* not very clearly with the "mass of a body," which he calls "*primary matter*;" force added renders it complete, and then it is "secondary matter." He traces the *origin* of our notion of force—rightly, we think—to ourselves as forces, the *moi* or *ego* being a self-conscious force, and giving us our first distinct notion of cause. Leibnitz is always more of a metaphysician (in the German sense) than of a psychologist—he reasons more than he observes; he is impatient of anything but results, or what he thinks are such; and he seems generally to care more to show what *must* be (as he supposes) than what *is*. Yet he did not fail to mark the psychological origin of the idea of force, as actually known in consciousness; and he justly makes self-consciousness our final appeal in all that concerns our mental history and experience,* for we know and feel that we are *causes*. But having greatly improved on the method of Descartes, by bringing prominently forward the idea of *force*, Leibnitz made a use of it which landed him in a theory as gratuitous as that of Descartes' vortices.

The *monads*, he tells us, were created all at once, by a single "fulguration" of the divine power, and will last for ever, if not annihilated by the same power. His argument for their being infinite in number is a curious example of the wildness, or at least hastiness, of some of his *à priori* reasonings. "It is possible that the monads should be infinite in number; and if they were not so, it would argue a defect in the divine resources; therefore they are thus infinite."† No two monads can be alike in all respects, for if they were, they would be identical; they must differ in the kind and degree of their inward activity.

* "De la Nature en elle-même. Considérations sur la Doctrine d'un Esprit universel," (first published in Erdmann's edition).

This is Leibnitz's "*principium identitatis indiscernibilium*," which Kant has so ably criticised.* These monads are not atoms of quantity (*atomi molis*), but of "substance"—"not material, but formal." They are forces, activities, souls (*âmes*.) The *materia prima* of body is merely passive and inert—it is not a complete substance till it is endowed with a form analogous to *soul*,—an original spontaneity of action, (ἐντελέχειαν τὴν πρώτην.) Our author even uses the word *spirit*, (*corpus constare ex materiâ et spiritu*.)† All these monads have more or less of perception and appetency. They are elsewhere described as metaphysical points, substantial forms, true unities, indivisible forces, without figure, without parts. Every created monad has its own activity always within itself; no other monad can originate any changes in it.

There are four sorts of monads. God is the primary monad (*monas monadum*). Next are finite spirits, including human souls, which are distinguished from the monads below them by reason, and the power of grasping necessary and eternal truth. Next in order are the souls of brutes; these have not reason, but only memory, perception, and volition. The lowest order of monads "possess merely the monadic life," a sort of impulsive force, without those faculties which even brutes possess; and this order includes vegetables and inorganic bodies. The essence of all *matter* consists of these monads of the lowest class; and even these are endowed by our imaginative philosopher with life and sensibility, as well as activity. They are all living, active beings, and are always struggling blindly to change their condition, and they have all some kind of "feeling or perception." Furthermore, one monad of the second order surrounded by an organized mass of monads of the lowest order, constitutes a human being. One monad of the third order surrounded by a similar aggregate, constitutes a brute animal. Though these monads are harmoniously accommodated to each other, they have no mutual influence or agency on each other; and mankind are deceived in the vulgar supposition that there is such a reciprocity of action always going on in the universe; this, as we shall presently see, was one great peculiarity of Leibnitz's system, leading to a further theory—that of pre-established harmony. Every monad, moreover, is a "mirror of the whole universe;" the whole universe is reflected in it, and is different from what it would be had any one single monad of the whole number not existed. As the monads have different degrees of perfection, so "each monad has a right to existence according to the perfection which belongs to it." An infinite number of these monadic universes were pos-

* Vid. "Reflexion's-begriffe. Kritik der rein. Vernunft."
† "Acta Erudit." 1698, pp. 434–6.

sible, but only one could exist; and the sufficient reason which determined the Deity to the choice of the actual one was, that it was the best. Hence another of Leibnitz's theories,—that of optimism.*

But enough of this ingenious philosophical romance—a speculation which seems to have originated very much in Leibnitz's anxiety to penetrate into the nature and all the relations of force, and was based on the strange and inconsistent notion that logical possibility led by a short road to truth. We need not wonder that the charge of darkness and mysticism should have been so widely brought against this extraordinary scheme, or that it should involve within itself many inconsistencies, and even contradictions—as when, in one place, he tells us that the monads of which matter is constituted are simple substances of the lowest order,† and elsewhere asserts that matter is not a substance, but a "*substantiatum*," which he explains by the expression, "well-founded phenomenon" (*phenomène bien fondé*);‡ language, this, which would seem much nearer to the subsequent German idealism than his reduction of the essence of matter to force.

Leibnitz's doctrine of monads involved that of PRE-ESTABLISHED HARMONY.§ The monads had no real agency on each other, but were mutually isolated and independent. Besides, "how could the higher monadic spirits act on the blind though living forces which constitute the lowest order of monads." This objection was as old as Empedocles, of the Italic sect—perhaps earlier. It was his maxim that "*like has no action on unlike*," a dogma which was fertile in producing the ancient idealism, founded on the supposed necessity of something intermediate in perception, holding both of object and subject. It is surprising that Leibnitz did not see the consequences of this doctrine, which, if legitimately carried out, would render even the divine agency in all created things impossible, for how could the infinite spirit act on materialism! But our sanguine theorist was very slow to realise the humiliating truth of the limitation of the human faculties, and to take it in connexion with another—namely, that "facts are stubborn things;" and he was still slower to make such considerations a main basis for philosophy. He totally denied that there was any reciprocal agency whatever in the universe, either between material things or between them and mind. The soul acts precisely as it would do if there were no bodies, and the body as it would if there were no souls. Leibnitz

* Vid. "Monad." 54. † Ibid. *passim.*
‡ "Lettre à M. Dangicourt."
§ Vid. "Monad.;" "De la Communication des Substances;" "La Nature en elle-même;" "Nouveau Système de la Nature," etc.

employs three hypotheses to account for the apparent agencies and harmonies of the universe. Two clocks exactly agree. This agreement may be supposed to arise from some reciprocal influence, or from the constant intervention of an artisan who regulates their respective movements every moment, or from the skill of the maker, who has so arranged the clocks at the beginning, that they cannot vary. The common theory with regard to soul and body is the first: they are supposed mutually to affect each other; the second theory—that of the continual intervention of the Creator—is the "*occasionalism*" of the Cartesians, termed by Descartes himself *assistentia* and *concursus;* the third, which Leibnitz adopts, is that of a harmony of all nature, pre-established by the Creator, and having the same effect as though there was the actual interagency which mankind imagine to take place between things. Our author rejected the first hypothesis on the Empedoclean principle; the second, as involving a perpetual miracle; and he adopted the third, as avoiding both difficulties, and as also best agreeing with another of his theories—namely, that there is always in the outward universe not only the same quantity of force, but also the same directions of motion. Descartes had said that there was always the same quantity of *actual* motion; this Leibnitz denied; but, as the monads were created all at once, "their forces, when summed up, would always be the same;" still, though apparently the soul could change the direction of motion at its pleasure, as Descartes had said, yet this variation, according to Leibnitz, could only take place by a pre-established harmony of all the directions of motion. Dr. Samuel Clarke, in his controversy with Leibnitz, evidently adopted the second hypothesis in preference to the third, though not, it would seem, to the exclusion of the first. The difficulty of drawing a line between the limit of divine agency and that of second causes (which Leibnitz, characteristically, seemed scarcely to appreciate) has induced modern writers of celebrity to maintain, without hesitation, that God must be supposed to be not only the author, and in some way the upholder, but the constant *actuator* of all the laws of matter and of mind; the only limit being in the moral agency of accountable beings, who are themselves *causes*, with an independent power of doing right and wrong.

Omitting other phases of Leibnitz's doctrine of pre-established harmony, we must yield to the temptation of quoting Dr. Thomas Brown's graphically popular, though somewhat exaggerated illustration of the main principle :

"The soul of Leibnitz would, though his body had been annihilated at birth, have felt and acted as if with its bodily appendage,—studying the same works, inventing the same systems, and carrying on, with the same warfare of books and epistles, the same long course of indefatigable controversy; and the body of this great philosopher, though his *soul* had been annihilated at birth, would not merely have gone through the same process of growth, eating and digesting, and performing all its other ordinary functions; but would have achieved for itself the same intellectual glory, without any consciousness of the works which it was writing and correcting,—would have argued with equal strenuousness for the principle of the sufficient reason, claimed the honour of the differential calculus, and laboured to prove this very system of the pre-established harmony, of which it would certainly in that case have been one of the most illustrious examples."*

This ludicrous mode of treating the subject may at least serve to relieve a necessarily dry discussion; though we apprehend that Leibnitz himself, whose general good humour would most likely have made him laugh with the rest could he have read it, would have replied, very obviously, that there could be no "harmony" at all where one of the terms of correlation was supposed wanting.

Leibnitz's extension of his theory of pre-established harmony to the "kingdom of God," by which he means, in general, the moral world, led immediately to his OPTIMISM. Conceiving that *à priori* reasoning, as explained in his views already given regarding the nature and certainty of human knowledge, led by a direct road to truth, he found little difficulty in supposing that, in his "monadology," he had solved the question relating to the nature and essence of substances. The pre-established harmony easily accounted for all the dynamics of the universe, and the actions of animal and moral beings; and now the result of the whole is, among all that were possible, the *best possible world*—that which contains the "greatest possible reality, unity, and agreement of the manifold in all its parts." But as evil, physical and moral, is a great and mournful fact of this world, it was our author's aim to point out the harmony of evil with the doctrine of Optimism. His work entitled "Essais de Theodicée, sur la Bonté de Dieu, la Liberté de l'Homme, et l'Origine du Mal," was published in consequence of the discussions on those subjects in Bayle's Dictionary.† Bayle had asserted that philosophy and theology were at variance; nay, that even reason was inconsistent with itself; and that it was impossible to reconcile the evil which exists, with the idea of a wise, just, and good author and governor of the universe. The Queen of Prussia, Sophia Charlotte, a lady who substituted a taste for learning and philosophy for the frivolities with which most personages of her rank are content, was aware of Leibnitz's views on several articles of the "Dic-

* Lecture XXXI.

† "Dictionnaire Historique et Critique," 1697.

tionary," and she desired him to publish on the subject. Hence the "Theodicée," of which our account must be very brief. The leading idea is, that as this world is the world which God has chosen, it is, notwithstanding its sin and its misery, the best of all possible worlds. Whatever discrepancy may appear between philosophical and theological truth in any point of their comparison, it is not real; therefore there can be no actual collision between reason and revelation. There are many things which we cannot comprehend; but we may sustain them against objections, provided they do not, *à priori*, involve logical contradiction, and are based on credible evidence.

In treating of evil, Bayle and Leibnitz set out in opposite directions. The former boldly maintains that the world is imperfect, and cannot come from such a being as Christians understand God to be; and that the old Manichean doctrine of two opposite principles, one *good*, the other *evil*, offers the only solution. Leibnitz sets out from the idea of the Deity, and maintains that such a Being must have constituted the most perfect possible world. He distinguishes between metaphysical, moral, and physical evil. The first is mere imperfection, the second is sin, the third is suffering. The first is wholly inseparable from the creature, for a being that is in all respects perfect must be God: there must be this distinction between the Creator and all creatures. And as to moral and physical evil, had they been excluded from following on that which is purely metaphysical, more imperfection would have accrued than by their admission. "Everything was foreseen, and everything that would possibly happen contributed ideally something to the divine determination to make the whole real." "It is true," says Leibnitz, "one might imagine possible worlds without sin and without suffering—romances and Utopias; but these worlds would be inferior in good to ours, since God *has* chosen this world as it is."* He goes on to show that the creature, as necessarily subject to limitation, (metaphysical evil) "cannot know everything, and may err." "God is not the author of this evil, which consists originally in privation or negation." He "produces in the creature all that is positive and good; but imperfections and defects in action come from the original limitation of the creature as such."† Leibnitz maintains fully the freedom of man's actions, and that God's foreknowledge does not interfere with it. By a distinction between the "provisional" and the "absolute" will of God, our philosopher thinks he reconciles the permission of evil with supreme goodness. He further adds: "We must conclude that God wills all good *antecedently*, and the best *consequently* as an end: he wills physical evil sometimes

* "Theod." Par. I. 16. † Ibid. 30–33.

as a means; but he only wills to permit moral evil on account of the *sine quâ non* or hypothetical necessity which allies moral evil with optimism (le meilleur); therefore the *consequent* will of God, which has moral evil for its object, is only permissive." But we cannot pretend to do anything like justice to the learning, devout feeling, ingenuity, metaphysical acumen, and praiseworthy desire to vindicate religion from gainsayers, which are manifested in the "Theodicée;" we are obliged to confess, however, that the grand and ancient question which it involves, (πόθεν τὸ κακὸν,) the origin of evil, is left by Leibnitz, as far as we can see, just where he found it. Pfaff of Tübingen, indeed, endeavoured to prove that Leibnitz did not believe his own doctrines, but agreed really with Bayle. This we hold to be a libel wholly without foundation, entirely disproved by the sincere tone of the "Theodicée," and by our author's general character and writings.

For further light on some of the views of our author which we have introduced to the reader, we must refer him to the letters which passed between him and Dr. Clarke, and which originated in a communication from Leibnitz to the Princess of Wales of that day, in which he passed an unfavourable judgment on the existing state of philosophical opinions in England. The enlightened Princess handed over this letter to Dr. Clarke, and a controversy began respecting the agency of the Deity in the universe, the principle of the sufficient reason, time and space, and other points, including certain views of Newton and Locke. On most of these topics there was evidently not all the difference of opinion which at first sight appeared, due allowance being made for the difficulty of adjusting the meaning of terms, and the different points of view from which the same subject is often apt to be regarded by different individuals. Leibnitz himself was fond of trying to accommodate differences, when possible, in this way; he especially aimed to diminish the points in controversy between himself and Locke, and expressly asserted, and with justice, that, fundamentally, their differences on some important topics were much less than appeared.

We can only touch on a few remaining speculations of our indefatigable and prolific genius. It was an old axiom, *natura non operatur per saltum.* Leibnitz called this principle the *law of continuity.* It is this same law by which variable quantities, passing from one magnitude to another, go through all the intermediate magnitudes, without passing over any of them abruptly. Leibnitz not only applied this law universally to nature with Boscovich, he extended it beyond the province of mathematics and physics, to mind itself. He proves, by its means, that the soul can never wholly cease to think, even in a swoon or in sleep ; and that the souls of brutes cannot be so much unlike those of men as is supposed, because there cannot be *discontinuity* between the two. On the same principle, he tells us, there cannot be, in strictness, such a thing as the death of an animated being. Might he not have proved, by the same law, that the interval of being between the created and the Creating mind must necessarily be filled up by insensible gradations; so that there can be no chasm between the finite and the infinite mind? But what a contradiction! Leibnitz, however, did nothing by halves. He had a principle—a law—and it must be necessary and universal! Of his physical and mechanical speculations, some estimate may be formed, when we state that he retained the Cartesian vortices of subtile matter, with a plenum; while he admitted Newton's principle of gravitation, though he has nowhere harmonized the two systems. Again, on the other hand, we find him writing to Dr. Clarke, that "in the time of Mr. Boyle, and other great men of Charles the Second's reign, no one would have dared to broach such whims as some of Newton's."* Was it that the world was not large enough for two such great men? He even strangely pronounced that Newton's views respecting the necessity of the conservating or regulating agency of the Creator in the universe had an impious tendency, by arguing imperfection in his works. Our author also engaged in a controversy with the Cartesians respecting the measure of force; the former maintaining that it is proportional to the square of the velocity; the latter that it is as the velocity—a dispute which arose from the apparent difference in the numerical quantities of different effects. Laplace has ingeniously shown, by experiment, that we ought to consider force as directly proportional to the velocity of the moving body.

After all the gigantic labours of the mind of Leibnitz, as seen in his speculative writings, which contained so many fertile germs of thought, destined to be developed by his successors—it is unquestionably as a mathematician that his fame rests on the most solid basis. The controversy respecting the discovery of the *Differential Calculus* has been almost national; the Germans having been as anxious to award that honour to Leibnitz, as the English to Newton. Dr. Guhrauer, the most recent biographer of Leibnitz, contends that the two discoveries were different; but for this opinion there is no foundation. It seems now admitted by all competent and impartial inquirers, that the two distinguished men both came independently to the same result by somewhat different methods, Newton representing by the flow of a point at the extremity of a line, what Leibnitz

* "On n'aurait pas oser nous débiter des notions si creuses," 5ième Écrit de Leibnitz.

represented by successive increments. But, on this subject, and on the relative merits of the two philosophers in regard to their respective developments of the method, it is sufficient to refer to the brief statement of Professor De Morgan.*

As a metaphysician and moralist, Leibnitz is best represented in his "Nouveaux Essais," (in which he criticises Locke,) and in his "Theodicée"—works which all should well study who desire to come into immediate contact with the mind of this extraordinary man. The latter is his most complete work, and a signal monument of his learning and genius. In these, and in many of his more fragmentary writings, there is much that has given law to philosophical opinion in subsequent times; though it must be admitted that the illustrious author frequently attempted unsuccessfully to transcend the real limits of human thought, apparently without knowing it. His principle of "contradiction" is invulnerable throughout the whole domain of logical truth. We must, however, contend that he carried his other principle of the "sufficient reason" too far, as a solution of difficulties connected with our contemplation of the moral universe. His doctrine of the "identity of indiscernibles" has been ably criticised by Kant, and shown to be untenable.† His notions of *à priori* truth, as a guide to theory, were obviously extravagant; in proof of which it is enough to refer to many parts of the "Monadology," which, agreeably to the remark of Brucker, are so perplexed and obscure as to confound his most admiring disciples. In regard to a pre-established harmony of the universe, no doubt there is a sense in which this is true; for, as has been beautifully said—here, even "all discord is harmony not understood" by mortals. But of Leibnitz's views on this subject, so far as they are an attempt to explain the phenomena of perception, or to solve the mystery of the mind's intercourse with the body and the outward universe, it may safely be affirmed that no theory was ever more gratuitous or baseless; and it did not survive Wolf, his successor and expounder, who himself much restricted its applications.

We have already seen that our author's "Optimism" followed consecutively from his doctrine of monads, and of pre-established harmony. The idea was originally Plato's; but Leibnitz's theory is generally accused of being more fatalistic than the Grecian, though he himself would not admit it. In the "Theodicée" he makes use of an allegory from Laurentius Valla. It relates to Sextus Tarquinius, and the outrage which caused the expulsion of his family from Rome. Leibnitz takes up the allegory where

* "Differential Calculus," pp. 33, 34.

† "Der Satz des Nichtzuunterscheidenden, *u. s. w.*;" Vid. "Amphibolie der Reflexionsbegriffe," s. 229; "Kritik der rein. Vern." (Rosenkranz).

Valla leaves it, and makes Theodorus, the high-priest at Dodona, ask Jupiter why he had not given another *will* to Tarquin? Jupiter sends the priest to Athens, to consult Minerva, who shows him the palace of the Fates, in which there were representations of all possible worlds, each containing a Sextus Tarquinius, with a different *will*. In the last and best of these worlds, the high-priest sees Sextus, such as he is, with his existing will and propensities. Minerva is made to say:

"You see, it was not my father that made Sextus wicked. He was wicked from all eternity, and he was so voluntarily. Jupiter has only bestowed on him that existence which he could not refuse him in the best of all possible worlds. He only transferred him from the region of possible to that of actual beings. But what great events does the crime of Sextus draw after it—the liberty of Rome—a government fertile in civil and military virtues—an empire destined to conquer and civilise the earth."

Theodorus returns thanks to Minerva, and acknowledges the justice of Jupiter. This is truly Leibnitzian: but, ingenious as it may be, we suppose that our readers will think it far enough from being a special proof that the present is the "best of all possible worlds." We may add, here, that our author's avowed theory of moral necessity appears to have been essentially that subsequently maintained by the great American metaphysician and divine, President Edwards.

Religion, no doubt, compels the conclusion, that whatever the Supreme Being does or permits, is done or permitted for the wisest possible ends. This is probably the full extent of optimism that it is given to man to realise; and it satisfies devotion. But Leibnitz went further, and evidently regarded his "Theodicæa" as claiming to be a more specific solution of the origin of evil. We wish we could go more fully into his reasonings; but we should not, by so doing, throw any further light on the subject. The main idea is, that this must be the best possible constitution of the moral as well as physical universe, because God, as the perfect Being, could only have chosen such a world. Whatever general sense, we repeat, this statement is capable of, the difficulty consists in its application, in detail, to the phenomena and aspects of evil; nor could the subject even be entertained at all, without theological discussions hardly suited to our pages. After all, our author proves that evil in the universe is a *good*, only by the fact of its existence. As to any independent argument, this great genius has failed, like all others who have attempted the question; for his solution amounts, in reality, to a *petitio principii*. It offers no satisfactory answer to the many questions which might be asked as to a possible universe viewed in connexion with a power, a wisdom, and a benevolence to which everything must be regarded as possible, but a logical contradiction.

There can be no doubt that, with all his genius, Leibnitz was eminently deficient, as compared with Bacon, Newton, or Locke, in that practical, inductive (we had almost said English) habit of mind, which some branches of truth especially demand. He has even been accused of extreme credulity. He tells us of a dog which he had heard speak several French words; among others, *thé*, *caffé*, *chocolat*, and *assemblée*.* Dugald Stewart thinks that the dog's master imposed on Leibnitz by means of ventriloquism. One thing is certain—that his genius enabled him easily to frame theories; and, when once enamoured of them, he no less easily could find in them harmonies with the principle of contradiction, or the sufficient reason, or some other favourite axiom; and he was then ready to maintain them with a zeal and a profusion of learning and argument worthy of the most renowned leaders of the middle ages. However unfinished many of his projects were, he seems never to have abandoned the expectation of accomplishing them. This remark applies to his vast scheme of a universal language, to his calculating machine, and a variety of dynamical inventions. Bailly says of him:

"As daring as Descartes, as subtile as Bayle, perhaps less profound than Newton, and less cautious than Locke, but alone universal among all these great men, Leibnitz appears to have embraced the domain of reason in all its extent, and to have contributed the most to diffuse that philosophical spirit which constitutes the glory of the present age."†

We close with a passage not less appropriate, from Miéville. The allusion is to the monument of Leibnitz, at Hanover, inscribed with the words *Ossa Leibnitzii:*

"Approach this tomb, and contemplate the man whom it contains. Observe the works deposited with him—his writings on theology and metaphysics, his letters on toleration, his profound researches on international law, the mass of his physical and mathematical solutions, and a variety of other intricate disquisitions, which combined to give him the character of the most general scholar of his age. He had the honour of sharing the invention of the Differential Calculus with the immortal Newton. An historian, a civilian, a metaphysician, and a poet, Leibnitz may be said to have embraced everything. The treasures of ancient learning were his, and he had the ambition to attempt a knowledge of the most abstruse subjects. He was thus led into bold speculations, from the pursuit of which he was sometimes recalled by the

* Vid. "Rapport de l'Académie Royale des Sciences, à Paris," 1706.

† "Eloge de Leibnitz."

admonitory lessons of history, while, at other times, he ventured beyond his powers, and allowed the guiding-thread to escape from his hold, but proceeded, unconscious of his loss, and bewildered himself in the illusions of system. He then no longer argued—an ardent imagination created for him an assemblage of fantastic beings; dazzling hypotheses deceived his reason; and when he hoped to succeed in laying open one labyrinth, he was entangled in another."*

